# Insight into the adsorption of a liquid organic hydrogen carrier, perhydro-*i*-dibenzyltoluene (*i* = *m*, *o*, *p*), on Pt, Pd and PtPd planar surfaces†

**DOI:** 10.1039/c8ra05800h

**Published:** 2018-09-12

**Authors:** Cecil Naphtaly Moro Ouma, Phillimon Modisha, Dmitri Bessarabov

**Affiliations:** HySA Infrastructure Centre of Competence, Faculty of Engineering, North-West University (NWU) P. Bag X6001 Potchefstroom 2520 South Africa Moro.Ouma@nwu.ac.za moronaphtaly84@gmail.com

## Abstract

Liquid organic hydrogen carriers (LOHCs) are considered to be safe and efficient hydrogen storage media with high hydrogen storage capacities. Adsorption of the LOHC perhydro-*i*-dibenzyltoluene (*i* = *meta* (*m*), *ortho* (*o*), *para* (*p*)) isomers on (100), (110) and (111) planar surfaces of Pd, Pt and a 50 : 50 PtPd alloy were investigated, using density functional theory with van der Waals corrections. The calculated heats of formation of the isomers indicated that all the isomers considered were energetically stable. Surface selectivity to isomer adsorption was investigated, using isomer adsorption preference and energies. The (110) surface was found to be highly preferred by the different isomers, compared with both the (100) and the (111) surfaces. Among the isomers, isomer–surface attachment occurred most often in the case of perhydro-*m*-dibenzyltoluene and perhydro-*o*-dibenzyltoluene adsorption. The LOHC isomer adsorption on different surfaces was found to be spontaneous, energetically stable and exothermic, with high isomer adsorption preference for Pt and PtPd surfaces, compared with Pd surfaces. This indicates the ease of loading of the LOHC on Pt and PtPd surfaces, for subsequent dehydrogenation.

## Abstract 2

Liquid organic hydrogen carrier (LOHC) interaction with a planar surface of a catalyst.

## Graphical abstract

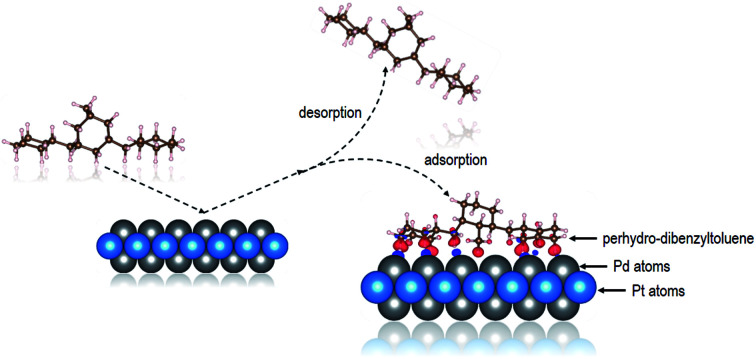

## Introduction

In ongoing efforts towards the goal of achieving sustainable carbon-free energy solutions, hydrogen and its associated technologies are attracting significant attention.^1,2^ This is largely because sustainable energy solutions, more often than not, carry the following shortcomings. Generation: some renewable energy sources (RES) are intermittent.^2–10^ Cost: the initial cost of setting up RES is high.^11–15^ Storage: the most commonly used energy storage medium is batteries; however, with the increasing demand, especially for electric vehicles, mineral sources (such those of Li, which plays an integral role in batteries) are continually being depleted, hence leading to increasing production costs.^1,11,12,15–17^ Transportation: transportation is closely associated with issues of storage; safe transportation requires safe energy storage technologies.^3,7,12,15,18^ Integration: the current energy infrastructure is grid-based and an integration of RES to the grid, so as to utilize the excess energies generated, is challenging.^19–22^ Toxicity: some of the elemental materials used, or by-products, are toxic.^16,23–25^

Because of these challenges, there is great need for a sustainable energy solution that will not suffer from the shortcomings mentioned (cost, storage, transportation, integration and toxicity). Hydrogen and its associated technologies is one such solution.^1,2^ This is because hydrogen can be generated from both renewable energy sources and fossil fuels,^15,17,26–29^ it is a clean energy carrier,^14,23,30^ and it can be used for both stationary and mobile power applications (*e.g.*, H_2_ fuel cell vehicles.)^9,23,30–32^

However, despite marked improvements in both hydrogen utilization and production, the application of hydrogen-based technologies in the current market landscape is hampered by its storage and delivery.^13,30^ Some of the proposed hydrogen storage solutions include physisorption of hydrogen in porous material surfaces, chemisorption of hydrogen on metal and metal alloy surfaces and the use of compressed hydrogen and cryogenic hydrogen.^30^ However, these proposed solutions still present challenges.

For onboard vehicular applications, liquid organic hydrogen carriers (LOHCs) are proposed to be a safe and efficient way to store hydrogen because of the reversible hydrogenation and dehydrogenation processes that can be activated using a catalyst.^19,30,33–38^ Hydrogen storage in these carriers takes place when the hydrogen covalently binds onto liquid organic compounds that are unsaturated/aromatic (hydrogen lean) in the presence of a catalyst until it is fully saturated/aliphatic (hydrogen rich). This process is referred to as hydrogenation of the liquid carrier. It is then possible to safely transport the hydrogen-rich liquid organic compound. The hydrogen molecule will then be released from the hydrogen-saturated liquid compound, also in the presence of catalyst, resulting in a hydrogen-lean liquid compound that can be hydrogenated once again. This process is referred to as dehydrogenation. It is this operating principle of LOHC systems that makes possible its integration in the existing fuel delivery infrastructure.^33,34,39–42^

**Fig. 1:**
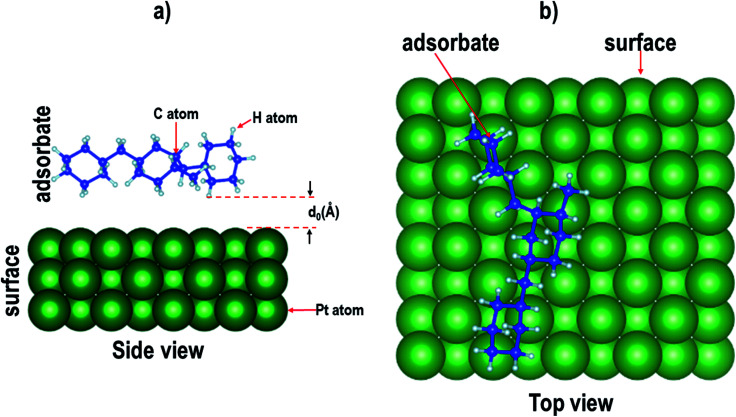
(a) Top view and (b) side view of the relaxed atomic structures of perhydro-*i*-dibenzyltoluene (*i* = *m*, *o*, *p*) derivatives. (The blue and grey balls indicate C and H atoms, respectively.)

Several materials have been proposed as possible LOHC candidates. Each has its own hydrogen storage capacity and operates at different (de)hydrogenation temperatures, over different catalysts.^10,23^ In this study, the fully hydrogenated (hydrogen-rich) LOHC, perhydro-dibenzyltoluene is investigated.^39,43^ Perhydro-dibenzyltoluene (chemical formula C_21_H_38_) is marketed by Sasol Performance Chemicals (South Africa) under the name Marlohc. Its derivative, Marlotherm SH (dibenzyltoluene), is used as a heat transfer fluid for many industrial applications where indirect heating is required.^36,41^ The most favourable physicochemical properties of dibenzyltoluene include high hydrogen storage capacity (6.2 wt%, 57 kg m^−3^), low melting point (−39 °C) and high boiling point (390 °C), and therefore low vapour pressure. The properties of perhydro-dibenzyltoluene as a candidate LOHC have been intensively explored experimentally.^3,6,36–39,41,44–46^ However, unlike other LOHCs,^5,19,23,40,47–52^ it has not yet been intensively explored theoretically.^39,41^ Perhydro-dibenzyltoluene exists in different isomeric forms with different isomer configurations.^38,39,41^ Its isomer configurations can be classified into perhydro-*i*-dibenzyltoluene, where *i* = *meta* (*m*), *ortho* (*o*) or *para* (*p*) (see Fig. 1).

LOHC (de)hydrogenation involves catalytic processes, which often take place on the surface of a catalyst. Hence, for (de)hydrogenation processes to take place, the reactants, either with or without some of the products of these processes, must bind to the catalyst surface. Pt is known to outperform other heterogeneous catalysts when it comes to catalytic activity, therefore it is an obvious choice for use in various applications.^53–57^ However, as Pt resources in the Earth's crust are decreasing, the cost of Pt is likely to increase.^57^ Alternatives to Pt, such as Pt-derived and non-Pt-based catalysts, are therefore now being explored—focus is being directed towards other transition metals.^42,53,58–62^ This is because the d orbital in transition metal elements affords a space for interesting catalysis chemistry as it determines the electronic properties of the element. For example, transition metals are known to be insulating, semiconducting and even conducting, depending on the filling of their d orbitals,^58,59,63–65^ and this is likely to determine the catalytic activity of the respective elements.^55,57,66,67^

Understanding the adsorption behaviour of a molecule (reactant/product) on a catalyst's surface is key in determining how a particular catalytic reaction will evolve.^59,68–70^ This has been shown in the case of H_2_, CH_4_, CO, H_2_S and NH_3_ adsorption on transition metal heterogeneous catalysts, bimetallic transition metal alloy catalysts and transition metal dopants, where the activity of the different catalysts was predicted using the adsorption (free) energy as a descriptor.^55,57,66,67,71,72^

In this study, the properties of perhydro-*i*-dibenzyltoluene (*i* = *o*, *m*, *p*) isomers adsorbed on planar ((100), (110) and (111)) surfaces of Pd, Pt and a 50 : 50 PtPd alloy were investigated, as the first step that will likely determine whether catalytic dehydrogenation of perhydro-*i*-dibenzyltoluene (*i* = *o*, *m*, *p*) will occur on the surface of the catalyst. (Dehydrogenation kinetics and mechanisms fall beyond the scope of this study.) All the proposed isomer configurations of perhydro-*i*-dibenzyltoluene (*i* = *o*, *m*, *p*)^39,43^ (see Fig. 1) were considered here. Furthermore, the surface selectivity towards isomer adsorption preference was then determined based on how many different isomers individually attached to a particular surface and also which particular isomer adsorbed on all the different surfaces.

## Computational details

Electronic structure calculations based on plane-augmented basis sets within the density functional theory formalism as implemented in the Quantum ESPRESSO code^73^ were used in this study. The exchange-correflation approximation was approximated using the generalized gradient approximation as parameterized by Perdew–Burke–Ernzerhof (PBE).^74^ Ultrasoft pseudopotential approximation was used to describe the valence electrons of Pd, Pt, C and H, and the electronic configurations of the elements were Kr 4d^10^, [Xe] 4f^14^ 5d^9^ 6s^1^, [He] 2s^2^ 2p^2^ and 1s^1^ for Pd, Pt, C and H, respectively. To account for the weak molecule–surface interactions, dispersion corrections were done using the vdW-DF2-B86R (rev-vdw-df2) scheme of van der Waals interactions implemented in the Quantum ESPRESSO code.^73,75^ Bulk unit cells of Pd, Pd and PtPd were first optimized to obtain their respective equilibrium properties. This was done using a kinetic energy cut-off of 30 Ry. The Brillouin zone (BZ) was sampled using a 10 × 10 × 10 *k*-point mesh with a 10^−6^ Ry and 10^−5^ Ry per Å convergence criterion imposed on the calculated total energies and forces, respectively. The obtained equilibrium lattice constants for Pd, Pt and PtPd were 3.95 Å, 3.98 Å and 3.98 Å, respectively. The different perhydro-*i*-dibenzyltoluene (*i* = *o*, *m*, *p*) isomer configurations were then relaxed using the cluster approach by embedding them in a 20 × 20 × 20 Å simulation box. For these systems, all the atomic positions were allowed to move. The only difference between these calculations and those of the bulk unit cells of Pd, Pt and PtPd was that Γ-sampling was used to sample the BZ. The relaxed perhydro-*i*-dibenzyltoluene (*i* = *o*, *m*, *p*) isomer configurations are presented in Fig. 1.

From the obtained equilibrium Pd, Pt and PtPd bulk structures, the (100), (110) and (111) surfaces consisting of three layers and 20 Å vacuum distances were constructed with only the bottom layer being fixed. The adsorption surface area (length × width) was chosen based on the full length of the relaxed adsorbate. This was done to ensure that the adsorbate is not strained by the surface size, its orientation on the surface, nor its interaction with its periodic image. The length and width of the surface was therefore calculated as *L*_surf_ = *L*_ads_ + 4 Å and *W*_surf_ = *W*_ads_ + 4 Å, respectively, where *L*_ads_ and *W*_ads_ are both equal to the longest isomer's horizontal length among the relaxed perhydro-*i*-dibenzyltoluene (*i* = *o*, *m*, *p*) isomer configurations. A further 4 Å was added to the adsorbate length (width) to possibly prevent adsorbate–adsorbate interaction with the periodic images of adsorbates. It should be noted, however, that the role of short-range and long-range adsorbate–adsorbate interaction was not part of this study, hence was not tested; the 4 Å addition is only an estimate.

**Fig. 2:**
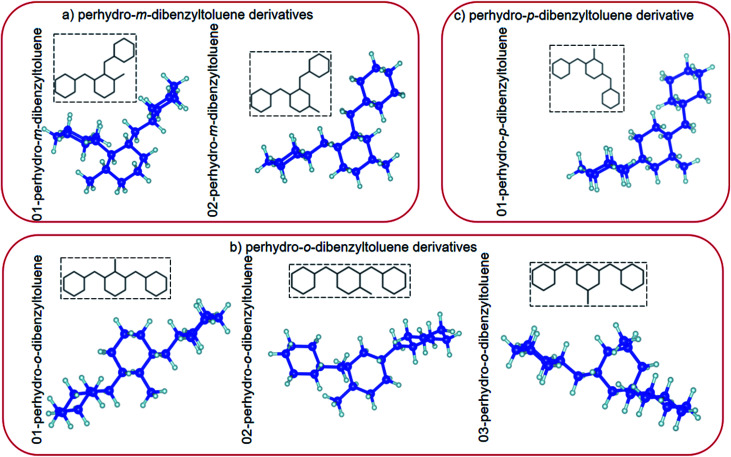
Schematic representation of the adsorption of an isomer of perhydro-dibenzyltoluene on a catalyst surface, where *d*_0_ is the equilibrium distance between the surface and the isomer. (The green, blue and grey balls represents Pt, C and H atoms, respectively.)

Nine surfaces were considered: each species (Pd, Pt and PtPd) had three planar surfaces ((100), (110) and (111)). Six isomer configurations were considered. See Fig. 1. Therefore, the adsorption of each of the isomers on each of the surfaces would require 54 calculations. Furthermore, as Fig. 2 shows, it is possible for each of the isomers to assume several adsorption configurations/orientations on the surface.

Thus, to account for all possible orientations on the different surfaces, a computationally less expensive approach was adopted. This was done using the adsorption locator module within Materials Studio.^76^ The adsorption locator module uses molecular dynamics (MD; with universal force fields) as the calculation engine of adsorption energies for all the generated surface–adsorbate configurations. The minimum energy surface–adsorbate configurations of a surface–adsorbate system are generated *via* a Monte Carlo search of the configurational space of the surface–adsorbate system, which includes different adsorbate rotations and orientations. At the end of the search, a minimum energy configuration as well as the preferred adsorbate adsorption site are obtained.

To validate this approach, of selecting the minimum energy configuration obtained using MD so as to determine whether it is consistent with vdW-DFT, a test case was carried out using ten different configurations generated using the adsorption locator module. These configurations were then used as inputs for vdW-DFT calculations. Atomic relaxations were done on the different configurations and adsorption energies were calculated. The calculated adsorption energies were then compared to those obtained using MD results. There was consistency between the MD and the vdW-DFT results in the prediction of the minimum energy configuration. See Fig. S1 in the ESI.† This observation is similar to that reported for the adsorption of natural organic matter on nanoparticle surfaces.^77^ Thus, for each isomer, only the configurations where the isomer attached to the surface (as predicted using the adsorption locator) were considered as inputs for subsequent DFT calculations. Configurations where the isomer did not attach to the surface were characterized by large values of the surface–adsorbate distance, *d*_0_ (see Fig. 2).

## Results and discussion

### Heats of formation

After relaxing the atomic positions of the perhydro-*i*-dibenzyltoluene (*i* = *o*, *m*, *p*) isomer configurations, the heats of formation of the respective isomer configurations were calculated as follows:

1 *E*_f_ = *E*_C_21_H_38__ − (21*E*_C_ + 38*E*_H_)

where *E*_C_21_H_38__ is the calculated total energy of the isomer, and *E*_C_ and *E*_H_ are the chemical potentials of C and H, respectively. This was done to confirm whether all the isomer configurations considered in this study would indeed form. The calculated heats of formation were negative for all the isomer configurations (Table 1), which indicated that all the proposed perhydro-*i*-dibenzyltoluene (*i* = *o*, *m*, *p*) isomer configurations would form. This observation is consistent with experimental observations, reported by Do *et al.*^39^ Perhydro-dibenzyltoluene is known to consist as a mixture of different isomers.^38,39,78^

**Table 1:** Heats of formation *E*_f_ of the different ionomers determined using DFT calculations with van der Waals corrections

Isomer configuration	*E*_f_ (eV)	*E*_f_ (kJ mol^−1^)
01 Perhydro-*m*-dibenzyltoluene	−8.01	−772.39
02 Perhydro-*m*-dibenzyltoluene	−8.03	−774.52
01 Perhydro-*o*-dibenzyltoluene	−8.03	−774.96
02 Perhydro-*o*-dibenzyltoluene	−8.02	−773.61
03 Perhydro-*o*-dibenzyltoluene	−7.95	−767.01
01 Perhydro-*p*-dibenzyltoluene	−8.12	−783.29

### Surface–isomer configurations

As already mentioned, only the configurations where the isomer attached to the surface were considered in subsequent DFT calculations. Table 2 shows that isomer attachment was only observed in 21 cases of the 54 surface isomer configuration searched. The (110) surface was highly preferred for perhydro-*i*-dibenzyltoluene (*i* = *o*, *m*, *p*) adsorption, with the exception of 03 perhydro-*o*-dibenzyltoluene and 01 perhydro-*p*-dibenzyltoluene on Pd (110) surfaces. The least preferred isomer adsorption surface was the (111) surface; isomer attachment occurred only in the case of 01 perhydro-*m*-dibenzyltoluene on Pd (111) and PtPd (111) surfaces, and 01 perhydro-*o*-dibenzyltoluene and 02 perhydro-*o*-dibenzyltoluene on Pd (111) and PtPd (111), respectively. The deduced order of surface preference by the different isomers was the (110) surface, followed by the (100) surface and then the (111) surface. Perhydro-*o*-dibenzyltoluene only attached to the PtPd (111) surface. Furthermore, the Pt and Pt-derived (PtPd) surfaces were preferred by the different perhydro-*i*-dibenzyltoluene (*i* = *o*, *m*, *p*) isomers to the Pd surface.

**Table 2:** Isomer attachment to Pt, Pt and PtPd surfaces (x indicates that the isomer attached to the particular surface)

Isomer configuration	Species	Surface		
		100	110	111
01 Perhydro-*m*-dibenzyltoluene	Pd	x	x	x
	Pt	x	x	
	PtPd		x	x
02 Perhydro-*m*-dibenzyltoluene	Pd		x	
	Pt	x		
	PtPd	x	x	
01 Perhydro-*o*-dibenzyltoluene	Pd			x
	Pt	x	x	
	PtPd		x	
02 Perhydro-*o*-dibenzyltoluene	Pd		x	
	Pt	x	x	
	PtPd		x	
03 Perhydro-*o*-dibenzyltoluene	Pd			
	Pt		x	
	PtPd		x	
01 Perhydro-*p*-dibenzyltoluene	Pd			
	Pt			
	PtPd		x	

This indicated that Pt doping/alloying might be essential in activating Pd surfaces for isomer adsorption

### Adsorption geometry relaxations

**Fig. 3:**
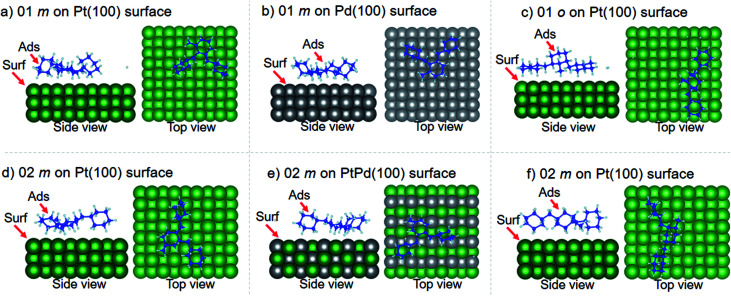
Obtained minimum energy for surface isomer configurations on (100) surfaces. (*m*: perhydro-*m*-dibenzyltoluene; *o*: perhydro-*o*-dibenzyltoluene.) – here and elsewhere.

**Fig. 4:**
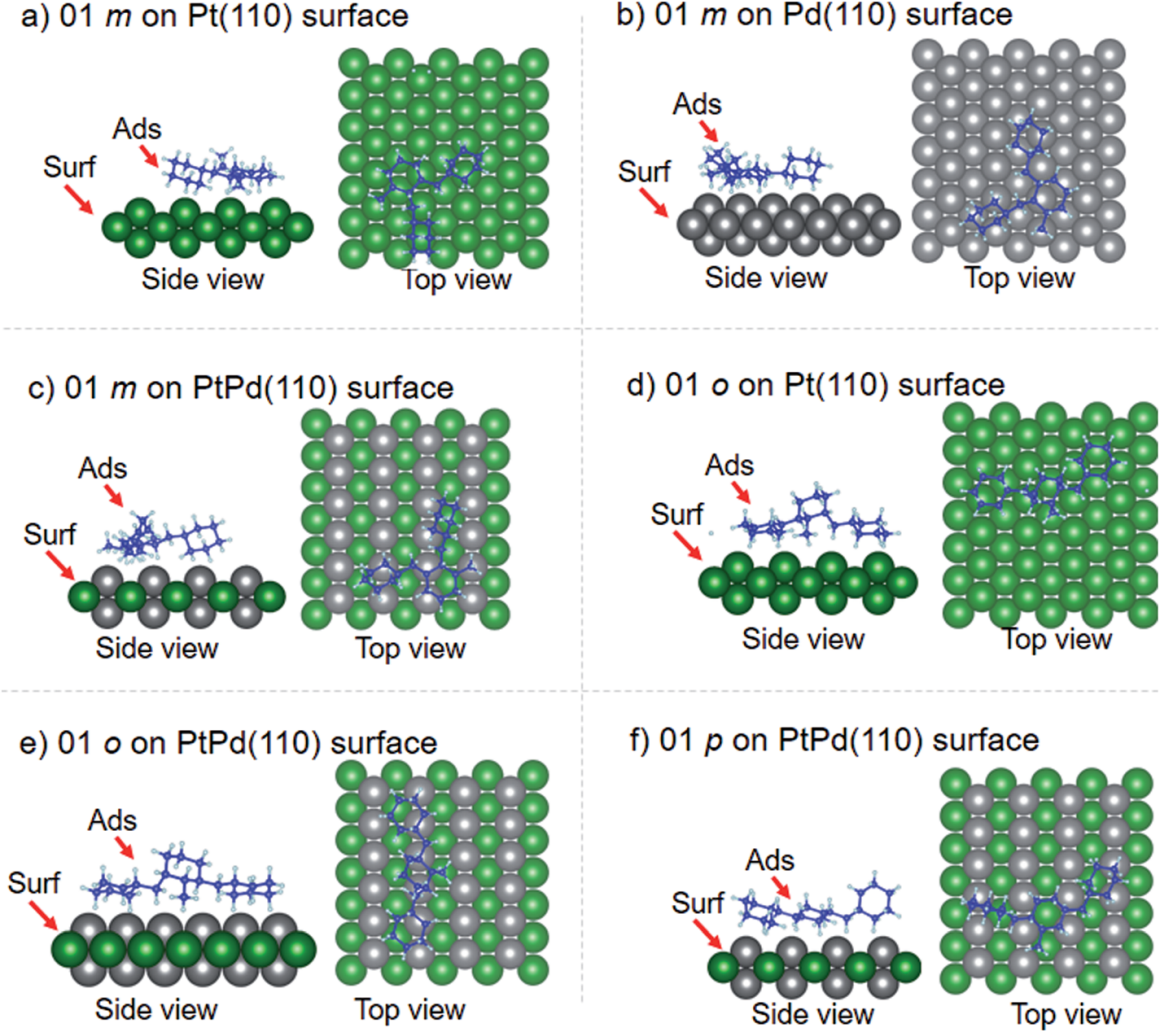
Obtained minimum energy for surface isomer configurations on (110) surfaces.

**Fig. 5:**
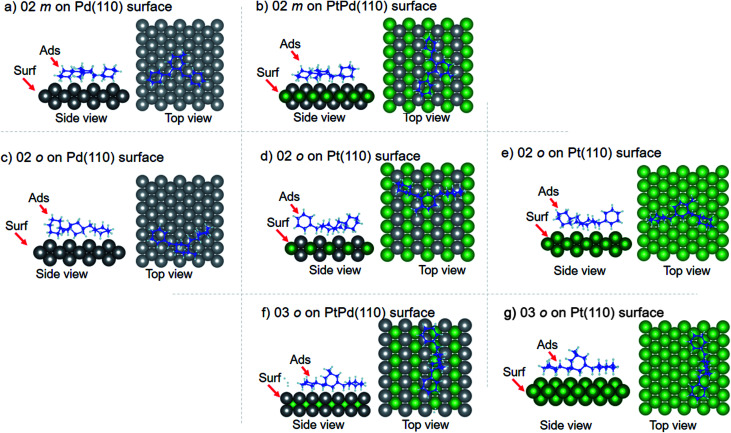
Obtained minimum energy surface for isomer configurations on (110) surfaces.

**Fig. 6:**
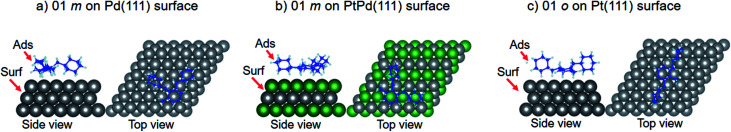
Obtained minimum energy for surface isomer configurations on (111) surfaces.

Using DFT calculations with van der Waals corrections, the 21 configurations (above) were then relaxed by allowing all atoms to move, with the exception of the atoms on the bottom layer of the surfaces. The relaxed surface–isomer configurations for the (100), (110) and (111) surfaces are shown in Fig. 3–6. In the case of isomer adsorption on (100) surfaces, the toluene lies horizontal to the surface, except in the cases of 01 perhydro-*o*-dibenzyltoluene and 02 perhydro-*m*-dibenzyltoluene adsorption on the Pt (100) surface. The toluene also lies horizontally on the surface in the case of isomer adsorption on (111) surfaces. In the case of (110) surfaces, the following were the exceptions: 01 perhydro-*o*-dibenzyltoluene on Pt (110) and PtPd (110) surfaces, 02 perhydro-*o*-dibenzyltoluene on the Pd (110) surface and 03 perhydro-*o*-dibenzyltoluene on Pt (110) and PtPd (110) surfaces.

The calculated equilibrium distances (*d*_0_) between the isomers and the different surfaces are tabulated in Table 3. For a particular isomer adsorption, the calculated *d*_0_ values revealed that Pd had the smallest *d*_0_ and Pt the largest. Some of the H atoms on the two benzene rings of the perhydro-*i*-dibenzyltoluene (*i* = *o*, *m*, *p*) isomers were found to be close to the metal surfaces and the change in C–H bond length for these H atoms was found to be increased by about 5%. This indicated that it would be easy to break these C–H bonds and liberate the H from these benzene rings than the toluene ring. This is consistent with experimental observations.^39^ A similar observation on C–H bond breaking and H liberation has also been reported in the case of dodecahydro-*N*-ethylcarbazole adsorption and dehydrogenation on Pd surfaces.^51,52^

**Table 3:** Equilibrium distance (*d*_0_) and adsorption energies (*E*_ads_) of perhydro-*i*-dibenzyltoluene (*i* = *o*, *m*, *p*) on (100) and (111) surfaces determined using van der Waals calculations

Isomer configuration	Species	Surface	*d*_0_ (Å)	*E*_ads_ (eV)	*E*_ads_ (kJ mol^−1^)
01 Perhydro-*m*-dibenzyltoluene	Pd	100	2.00	−1.90	−183.06
	Pt	100	2.28	−1.62	−156.05
02 Perhydro-*m*-dibenzyltoluene	Pt	100	1.98	−1.72	−166.43
	PtPd	100	1.96	−1.64	−158.61
01 Perhydro-*o*-dibenzyltoluene	Pt	100	2.18	−1.55	−149.75
01 Perhydro-*o*-dibenzyltoluene	Pt	100	2.25	−1.35	−129.79
01 Perhydro-*m*-dibenzyltoluene	Pd	110	1.49	−2.45	−236.52
	Pt	110	1.75	−2.89	−279.21
	PtPd	110	1.59	−2.29	−221.07
02 Perhydro-*m*-dibenzyltoluene	Pd	110	1.73	−2.69	−259.98
	PtPd	110	1.42	−2.45	−236.40
01 Perhydro-*o*-dibenzyltoluene	Pt	110	1.80	−2.28	−219.82
	PtPd	110	1.53	−2.40	−231.57
02 Perhydro-*o*-dibenzyltoluene	Pd	110	1.44	−2.37	−228.22
	Pt	110	1.61	−2.20	−212.62
	PtPd	110	1.50	−2.22	−214.05
03 Perhydro-*o*-dibenzyltoluene	Pt	110	1.69	−2.63	−253.86
	PtPd	110	1.53	−2.57	−248.45
01 Perhydro-*p*-dibenzyltoluene	PtPd	110	1.53	−2.23	−214.84
01 Perhydro-*m*-dibenzyltoluene	Pd	111	1.90	−2.08	−201.04
	PtPd	111	2.15	−1.86	−179.38
01 Perhydro-*o*-dibenzyltoluene	Pt	111	1.79	−2.02	−194.98

### Adsorption energy

Furthermore, since in hydrogen evolution reactions (HERs), the adsorption free energy of a molecule, Δ*G*_M_, has been used as a descriptor for HER activity, it is evident that *E*_ads_ can then also be used to estimate the HER activity of a surface. The following applies to the *G*_M_:

2 Δ*G*_M_ = *E*_ads_ + Δ*E*_ZPE_ + *T*Δ*S*_M_

where Δ*E*_ZPE_ is the zero point energy difference between the molecule M in the adsorbed and gas phase, Δ*S*_M_ is the change in entropy between the molecule M in the adsorbed and gas phase, and *T* is the temperature in K. In the case of hydrogen, *E*_ads_ ≅ 0.24 eV.^57,71,72^ Pt is considered as the optimal catalyst for HER because its Δ*G*_H_ ≅ 0 eV, and hence thermoneutral. Assuming Pt to be the best catalyst for isomer adsorption in this study, the energy difference from Pt values was then estimated as follows:

3 Δ*E*_TN_ = *E*^Pd(PtPd)^_ads_ − *E*^Pt^_ads_

where *E*^Pd(PtPd)^_ads_ and *E*^Pt^_ads_ are the calculated isomer adsorption energies (obtained using eqn (2)) on Pd or PtPd and Pt surfaces, respectively. Table 4 shows that Δ*E*_TN_ < ±0.1 eV in the case of isomer adsorption on PtPd surfaces and Δ*E*_TN_ > ±0.1 eV in the case of isomer adsorption on Pd surfaces. Thus, alloying Pd and Pt is likely to improve the activity of Pd towards isomer adsorption and probably also the catalytic activity.

**Table 4:** Activity estimation using Δ*E*_TN_

Isomer configuration	Species	Surface	Δ*E*_TN_ (eV)
02 Perhydro-*m*-dibenzyltoluene	Pt	100	0.00
	PtPd	100	0.08
01 Perhydro-*m*-dibenzyltoluene	Pd	110	0.44
	Pt	110	0.00
	PtPd	110	0.60
01 Perhydro-*o*-dibenzyltoluene	Pt	110	0.00
	PtPd	110	−0.12
02 Perhydro-*o*-dibenzyltoluene	Pd	110	−0.16
	Pt	110	0.00
	PtPd	110	−0.01
03 Perhydro-*o*-dibenzyltoluene	Pt	110	0.00
	PtPd	110	0.06

The catalytic activity of Pt and Pd on alumina supports has been investigated for the dehydrogenation of perhydro-dibenzyltoluene.^79^ It was discovered, experimentally, that 5 wt% Pt/Al_2_0_3_ has high activity (40% degree of dehydrogenation) compared with 5 wt% Pd/Al_2_O_3_ (8% degree of dehydrogenation). A compound chemically similar to perhydro-dibenzyltoluene, cyclohexane, showed similar behaviour, with even lower activity when bimetallic Pt–Pd was used for dehydrogenation.^42^ In the dehydrogenation of decalin to naphthalene, DFT calculations revealed the following. The first dehydrogenation step (decalin to tetralin) is energetically more favoured on Pt surfaces than on Pd surfaces, while the reverse applies for the second dehydrogenation step (tetralin to naphthalene). Using the estimate obtained using eqn (3), the activity of the Pt (110) surface towards dehydrogenation is more energetically favourable in the case of 01 perhydro-*m*-dibenzyltoluene, 02 perhydro-*m*-dibenzyltoluene and 03 perhydro-*o*-dibenzyltoluene than either the Pd (110) or PtPd (110) surfaces. This observation is similar to what is reported elsewhere in literature.^79^ In the case of 01 perhydro-*o*-dibenzyltoluene and 02 perhydro-*o*-dibenzyltoluene, dehydrogenation will be more energetically favoured on the Pd (110) and PtPd (110) surfaces that on the Pt (110) surface.

### Charge analysis

**Fig. 7:**
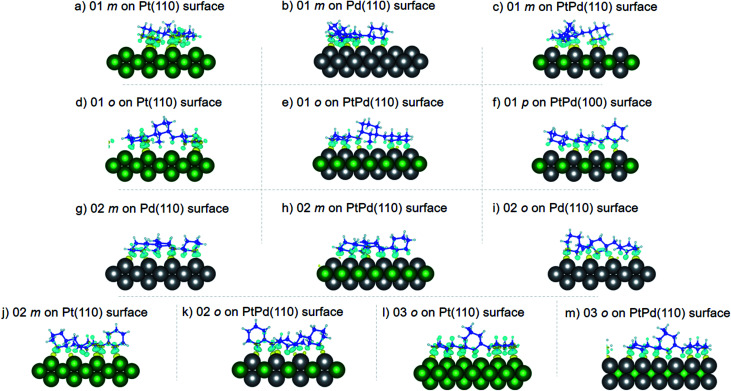
Obtained charge density difference for surface isomer configurations on (110) surfaces.

**Fig. 8:**
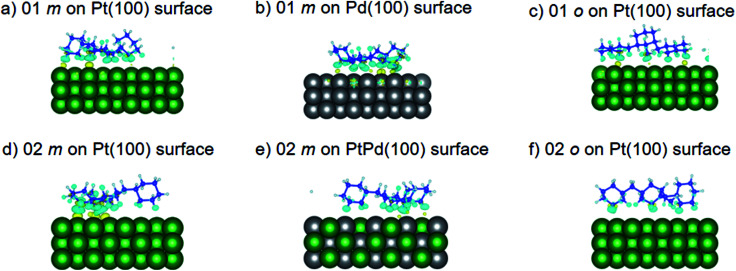
Obtained charge density difference for surface isomer configurations on (100) surfaces.

**Fig. 9:**
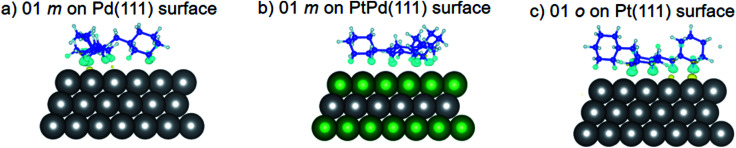
van der Waals DFT calculated charge density difference for surface isomer configurations on (111) surfaces.

Charge analysis was carried out in efforts to better understand how the charges are distributed in the isomer–adsorbate systems. Charge density difference was determined as follows:

4 *ρ*_CDD_ = *ρ*_IS_ − (*ρ*_I_ + *ρ*_S_)

where the charge density distribution of the isomer (*ρ*_I_) and the pristine surface (*ρ*_S_) were subtracted from the charge density distribution of the isomer–surface system (*ρ*_IS_). The obtained charge density difference (*ρ*_CDD_) at ±0.002 iso-surface levels are shown in Fig. 7–9. In the figures, yellow regions indicate regions where *ρ*_CDD_ is positive while the light blue regions indicate regions where *ρ*_CDD_ in negative. It is evident that charge accumulation occurs at the interface of the surface and the isomer, with negative charge accumulation largely concentrated on H atoms and C–H covalent bonds for the H atoms close to the surfaces. This indicated that there will be charge transfer between the isomer and the surface.

## Conclusions

Surface and isomer selectivity was investigated using DFT calculations with van der Waals corrections. Different isomer configurations of perhydro-*i*-dibenzyltoluene (*i* = *o*, *m*, *p*) on planar ((100), (110) and (111)) surfaces of Pd, Pt and PtPd were investigated. Although all the perhydro-*i*-dibenzyltoluene (*i* = *o*, *m*, *p*) isomers and their associated configurations were found to be stable, adsorption of the perhydro-*p*-dibenzyltoluene isomer and its configurations on the different surfaces was found to be least likely; perhydro-*i*-dibenzyltoluene (*i* = *o*, *m*) was much more likely to be adsorbed on the surfaces under consideration. Isomer adsorption on the (110) Pd, Pt and PtPd surfaces was highly preferred, followed by the (100) surfaces and the (111) surfaces. This indicates that, among the surfaces considered in this study, the best surface for catalytic reaction will be the (110) surfaces of Pd, Pt and PtPd. Pt surfaces had the highest adsorbate binding energies among the individual perhydro-*i*-dibenzyltoluene (*i* = *o*, *m*, *p*) isomer configurations, followed by the PtPd alloy and then, finally, the Pd surfaces.

## Conflicts of interest

There are no conflicts to declare.

## Supplementary Material
